# Affective and predatory violence: from evolutionary adaptation to psychiatric morbidity

**DOI:** 10.3389/fpsyt.2025.1690508

**Published:** 2025-09-24

**Authors:** J. Reid Meloy

**Affiliations:** San Diego Psychoanalytic Center, San Diego, CA, United States

**Keywords:** violence, affective violence, predatory violence, threat assessment, forensic examination

## Abstract

Nearly a century of mammalian research has supported the bimodal nature of violence. Predatory (instrumental) violence finds its evolutionary origins in hunting, while affective (reactive, impulsive) violence originates in the need to defend against an imminent threat. Both modes of violence serve survival, and none of us would be here if our ancestors did not excel at both. The capacity for both affective and predatory violence is neurobiologically atavistic, but contemporary society controls its expression through social learning, cultural guardrails and legal sanctions. Psychiatry and other mental health professions, however, often confront both affective and predatory violence in the context of psychiatric and personality disorders; and specifically, in their roles as threat assessors or forensic evaluators. This perspective underscores the importance of discerning extremely violent events as either affective or predatory, and details the criteria for doing so.

## Introduction

The human species has survived because of its capacity for both affective and predatory violence. The evolutionary adaptation of affective violence, also known as reactive or impulsive violence, was characterized by intense autonomic reactivity, usually accompanied by fear and a cascade of hormones, and culminated in a sudden physical defense against a perceived imminent threat. There was no time to contemplate whether the moving bush on the savannah was caused by the wind or a predator; the only recourse was the limbically-driven “low road” to either freeze, flee, or fight. On the other hand, survival also required food, and here contemplation had a role: the hunt had to be planned and purposeful, sometimes done with other members of the tribe, and the physiology was markedly different. Those who hunted with minimal autonomic arousal, no anger or fear, and took the “high road” by engaging their pre-frontal cortex to plan and prepare in advance, were likely to return with food for their hungry offspring. Those that did not or could not adapt to the rigors of stealth and clear-eyed attention while they hunted, eventually died of starvation and they did not genetically survive.

Over the course of thousands of years, our species has evolved wherein affective defense and predatory attack are rare events in most people’s lives. Social learning, cultural guardrails, and legal sanctions control the expression of affective or predatory violence, yet the neurobiology of these modes of violence is atavistic, and remains largely unchanged as a *capacity* within humans (1, 2).

On a frequent basis, however, psychiatrists, psychologists and other mental health professionals are called upon to assess those who engage in either affective violence, predatory violence, or both. Such evaluations are typically done in a forensic context to examine an act retrospectively; and now more often, in a threat assessment context wherein the risk of such acts is judged prospectively (3). In both cases, determining whether violence is affective or predatory is fundamental to such assessments, and shapes both the interpretation of data, as well as the prognosis and often diagnosis of the forensic patient or subject of concern (1).

### History

The provenance of research on affective and predatory violence began with the experimental study of cats in the early twentieth century (4). Affective violence was called “affective defense” (5), and observers noted that when threatened, cats behaved with distinctive behavioral markers: piloerection, arched back, extension of teeth and claws, dilated pupils, pinned back ears, and intense vocalization. Neurobiological correlates were also identified with a particular focus on the hypothalamus (6, 7). Predatory violence was first studied by Flynn and colleagues (8–10) and was defined both behaviorally and physiologically in cats by the *absence* of sympathetic activation: low profile to the ground, no teeth or claws during approach, no pupil dilation, no piloerection, no arched back, the use of ears for sound localization, and no vocalization. The stealth resulted in a swiftly executed and precise attack to the head of the prey.

Both experimental and quai-experimental animal research has continued to inform this model of aggression (4, 11) wherein the neuroanatomical structures and neurochemicals utilized are different (12). Scientific research in understanding this model’s relevance to humans has also validated certain findings over the past forty years: differential neurotransmitter activation dependent upon the mode of violence (13); differential psychopharmacological treatment of violent patients (14–16); differential structural and functional imaging of patients with histories of affective and/or predatory violence (17, 18); differences across various measures of neurocognitive functioning (19); differences in children and adolescents with histories of either proactive or reactive aggression, and their predictive value (20, 21); and the modes of violence in relationship to various diagnoses of mental and personality disorders (22–24).

Most pertinent to this Perspective, however, is the bimodal theory’s accurate application to patients who appear to have engaged in affective or predatory violence, or patients where concern regarding such future behavior is paramount. My contribution to this line of research has been the identification of normative criteria for determining whether the violent behavior in question is either affective or predatory (1, 25). Since first publication, validation of these criteria has been promising (24, Hanlon et al., 2013, 4, 17, 26). **Table 1** sets forth the ten criteria for determination of affective vs. predatory violence. These criteria were initially published nearly four decades ago (25), and have remained the same with the exception of slight modification of the ninth measure, Awareness (1, 26–30). The reader will notice that the criteria are generally binary, and when one criterion is evident in a case, the others are likely to follow given the concordance between physiological and behavioral research that underpins the model. However, research also indicates that this is a bimodal, not a categorical theory (31, 32).

**Table 1:** Comparison of Criteria for Affective and Predatory Violence (1, 25).

Affective violence	Predatory violence
1. Intense autonomic arousal	Minimal autonomic arousal
2. Subjective experience of emotion	No conscious emotion
3. Reactive and immediate violence	Planned and purposeful violence
4. Perceived threat	No imminent perceived threat
5. Goal is threat reduction	Variable goals
6. Possible displacement of target	No displacement of the target
7. Time-limited behavioral sequence	No time-limited sequence
8. Preceded by public posturing	Preceded by private ritual
9. Altered awareness	Focused awareness
10. Primarily emotional/defense	Primarily cognitive/attack

## Discussion

### The criteria

#### Intense autonomic arousal vs. minimal autonomic arousal

Affective violence in humans is preceded by intense sympathetic arousal, behaviorally evident in skin flush, muscular tension, rapid and shallow breathing, perspiration, and dilated pupils. In predatory violence, there are no somatic markers evident to the observer (33), which increases the danger risk since there is no attack signaling. Recent research has formulated and validated a typology of proximal warning behaviors for predation in humans, what is referred to as targeted violence, that is not dependent on any physiological arousal (3, 34).

#### Subjective experience of emotion vs. no conscious emotion

Affective violence is typically preceded by the emotions of anger and/or fear. It is a “low road” limbically-driven mode of violence. When an imminent threat is perceived by the amygdala, both “fast” catecholamines (adrenaline and noradrenaline) and “slow” cortisol, a glucocorticoid hormone, are released by the adrenal medulla and cortex, respectively. Receptor systems bind these hormones, and the hypothalamus triggers the HPA axis which both releases corticotropin-releasing hormone and activates the sympathetic nervous system. The prefrontal cortex will regulate the emotions of fear and anger, and modulate amygdala activity, while the hippocampus is involved in the contextual memory of fear and/or anger. In extreme cases of affective violence, the intensity of the emotion will result in the affect being split off and the perceptual experience of dissociation or depersonalization; the patient will report after the event that they felt separate or outside their body, and often have partial amnesia for the details—although this latter self report may be feigned in forensic cases.

In predation, given the predominance of “high road” pre-frontal cortical planning and preparation, there is minimal or no conscious experience of any emotion. Key neural structures in predatory violence will dynamically work together for target evaluation and approach behavior; these include the basolateral amygdala, the lateral hypothalamus, the dorsolateral gray (PAG), and the orbitofrontal prefrontal cortex to plan, inhibit emotion, and execute goal-driven actions. Key neurochemicals during predation include dopamine, endocannabinoids to modulate pain and affect, and the inhibiting gamma-aminobutyric acid (GABA) which likely blunts emotions during predation. The mass murderer, given the predatory mode of virtually all such cases, will self report in retrospect no felt emotion during his crimes. This original finding (35) has been subsequently validated by eye witness accounts of the calm, deliberate, and methodical behavior of targeted attackers over the past two decades (36). Such a finding, moreover, is supported by the evolutionary need for calmness and stealth while hunting to ensure success and an available food supply. Animal research has found through direct measures that emotionally suppressing neurotransmitters, such as GABA, are utilized during a predatory mode of violence (12, 15).

#### Reactive violence vs. planned, purposeful violence

Affective violence is a reaction to a perceived threat. There is no time delay as the HPA (hypothalamic-pituitary-adrenal) axis is mobilized and sudden defensive violence plays out when neither freezing or fleeing is an option. In predatory violence, there is engagement of the pre-frontal cortex to plan and prepare for the attack regardless of motivation. Time delays may range from hours, to days, months, or even years (25).

#### Perceived threat vs. no perceived threat

In affective violence there is always a perceived threat. It may be an actual external threat, such as a person or animal mounting an attack or an imminent threat by a stranger to one’s child; or it may be an internal threat that often has a psychiatric basis: panic anxiety, a phobia, fear of abandonment, the perception of humiliation, an auditory or visual hallucination, an intrusive recollection, or a myriad of possible delusional beliefs that portend a loss of control, often manifest as paranoid symptoms. There are countless possibilities when the threat is internally generated in the patient’s mind, often caused by a diagnosable medical or psychiatric condition.

#### Goal is threat reduction vs. variable goals

In affective violence, the goal is simply to reduce the threat. Violence is utilized to hurt, injure, or in extreme cases, kill the imminent threat. Such intent, when litigated in a criminal context and accepted by the trier of fact, will mitigate or even eliminate criminal responsibility for the defensive act of violence. In predatory violence, goals are numerous and variable, particularly in contemporary society: revenge, money, sexual gratification, physical or psychological dominance, territorial expansion, the elimination of a witness who poses a potential long term threat, ideological dominance of another group, religious mandate, or a nation-state conflict. The possibilities are myriad, but the fundamental evolutionary basis for the predation is not lost—hunting to hurt or kill the target. Special operators in the military are trained to hunt and kill on behalf of the nation. Psychopaths may be hard-wired to engage in predation given their biology and propensity to do so (26), but without the honor, loyalty, and willingness to fight and die of the soldier.

#### Possible displacement vs. no displacement of the target

In affective violence, the intense sympathetic arousal joined with a lack of pre-frontal cortical discernment risks the rapid shifting of violence from the original threat to a third party. This is practically evident in two situations: first, in inpatient psychiatric settings, the management of patient assaultive behavior, which is usually affective, precludes stepping in between the two warring patients to avoid an attack on the staff person attempting to intervene. Second, the most dangerous callout for law enforcement is domestic violence. Such violence is mostly affective, and the patrol officer is at great risk to be assaulted if he or she steps into the midst of the conflict.

#### Time limited vs. no time limitations

In affective violence, the intense sympathetic arousal, originally called the “alarm state” of the General Adaptation Syndrome (37) precludes defensive violence for more than seconds or minutes before fatigue and decrements in muscular coordination signal the body’s attempt to return to homeostasis. In predation, research, planning, and preparation are higher cortical tasks and not dependent on limbically-driven arousal; hence, time frames for predatory violence can range from days, weeks, to months, and even years. FBI research (36) indicates that 62% of a sample of active shooters spent 1–24 months planning their targeted attacks.

#### Preceded by public posturing vs. private ritual

In affective violence, the public posturing is often instinctual: loud vocalization, fist clenching, jaw clenching, posturing, staring, and expansion of the thorax to both oxygenate more efficiently and look as physically large and imposing as possible to the threat—all in the service of one goal, to make the threat go away. In predation, since stealth is central to success, there is no public posturing, but instead private ritual, often to enhance the narcissism of the predator. He may adorn himself with certain identity claims (38), such as tattoos, amulets, medallions, insignias, weapons and clothing that signal his predatory skill, burnishes his self esteem, or links him to the symbols of a larger cause or movement. Stalking or reconnaissance of the target may serve a tactical purpose, but also psychologically validates his control over the victim.

#### Altered awareness vs focused awareness

In affective violence, attention and awareness are often altered; research indicates that time will subjectively slow, sounds will be muted, tunnel vision will occur, dissociative states may be reported, and memory loss may be partial or complete for the event (39). In predation, there are suggestions of selection suppression of other stimuli and enhanced focus on the target, documented in self-reports, witness reports, and video capturing of individuals engaged in acts of mass murder (26, 35).

#### Emotional defense vs. cognitive attack

Although both the limbic system and pre-frontal cortical system are in dynamic interplay in any act of violence, a simple binary distinction is valuable. Affective violence is defensive in nature, and will often be characterized by the patient in retrospect as being “carried away,” or by the clinician as “out of character” for the patient. The intensity of emotion within affective violence reveals the primitive evolutionary roots of this survival behavior. On the other hand, the calmness, rationality, attention, and rehearsal fantasy evident in predation (26, 40) reveals the ancestral evolution of our species as successful hunters.

## Forensic and threat assessment applications

Retrospective use of the model has found application in both criminal and civil forensic litigation (1, 4, 26, 41), and both inpatient and outpatient management of the violent psychiatric patient when such a history is known (42, 43).

Both Eichelman (13, 16) and Siever (15) have contributed to the pharmacological management of the inpatient and outpatient psychiatric patient by highlighting the importance of differentiating between a history of affective and predatory violence. Medication management of the former has been much more successful. Forensic examination of a patient who has committed a violent act, especially in the context of an insanity plea, is often much more granular and accurate if the mode of violence is determined independent of the psychiatric diagnosis. Both modes of violence, of course, can be committed by a psychotic individual, and in cases of predation, there may be rational planning within the irrationality of a delusional belief system (44). Paradoxically, the delusions may bring a commitment to the act of predation that would otherwise be suffused with anxiety and ambivalence. From a more plebeian perspective, most acts of violence by a psychiatric patient are affective; however, in the adversarial arena of litigation, the forensic examiner should remain cognizant of the fact that defense attorneys will want to characterize most acts of violence as affective, and prosecutors will be biased toward all acts of violence as predatory.

Prospective use of the model appears to have predictive utility in both threat assessment and management of those at risk for targeted, predatory violence (3). Such psychiatric work is usually done within a threat assessment team, and the role of the psychiatrist is often to establish whether or not there is a nexus between the symptoms of the disorder, if present, and a motivation to be violent. Within the behavioral threat assessment context, concerning, observable behaviors *in the present* are most time sensitive to the task at hand: to mitigate risk. Ongoing management of the case, moreover, requires an accurate diagnosis and appropriate treatment planning. Diagnostic complexities abound in threat assessment, and a common error among psychiatrists is to ignore the additional presence of a personality disorder when a major mental disorder has already been diagnosed. For example, most salient is the finding that predatory violence and psychopathy in a psychiatric patient are closely related (45). As Blair et al (23) wrote, “No biologically based disorder other than psychopathy is associated with an increased risk of instrumental [predatory] aggression” (p. 155).

## Conclusion

The normative criteria for the bimodal classification of aggression and violence, introduced almost four decades ago, provide a reliable and valid methodology for the mental health clinician to both retrospectively and prospectively understand the psychobiology of the aggressive, and potentially dangerous patient (46). Accurate discernment of the mode of violence by psychiatrists and other mental health professionals is foundational for the forensic and treatment opinions and decisions which follow (30, 47). Further research, however, is warranted. There is very limited neuroimaging research (17, 18), and although quite promising, further validation of the differential functioning of the brain in subjects who have histories of affective or predatory violence has not been done. From a safety perspective, ranging from the inpatient unit to the community at large, pharmacological and other treatment intervention studies would be most helpful to mitigate risk. And extremism in all its forms gives rise, on occasion, to the violent signal which portends danger (48). As Freud wrote in 1930, “the inclination to aggression is an original, self-subsisting instinctual disposition in man, and I return to my view that it constitutes the greatest impediment to civilization” (p. 122).

## Data Availability

The original contributions presented in the study are included in the article/supplementary material, further inquiries can be directed to the corresponding author/s.
